# The impact of depression and cardiovascular disease on fall risk in type 2 diabetes mellitus: a gender and sleep status analysis

**DOI:** 10.3389/fpubh.2025.1488923

**Published:** 2025-03-19

**Authors:** Kehua Li, Xue Chen, Lang Chen, Dianyin Liang

**Affiliations:** ^1^Department of Physiology and Pathophysiology, Yulin Campus of Guangxi Medical University, Yulin, China; ^2^Department of Basic Medical Experiment Teaching Center, Yulin Campus of Guangxi Medical University, Yulin, China; ^3^Department of Stomatology, People's Hospital of Luchuan, Yulin, China; ^4^Department of Public Health, School of Medicine, Guangxi University of Science and Technology, Liuzhou, China

**Keywords:** depression, cardiovascular disease, type 2 diabetes, falls, additive interaction

## Abstract

**Background:**

This study aims to examine the combined effects of depressive symptoms (DS) and cardiovascular disease (CVD) on fall risk in patients with type 2 diabetes mellitus (T2DM), as well as evaluating the potential moderating effects of sleep status and gender.

**Methods:**

This study analyzed 941 participants from the China Health and Retirement Longitudinal Study (CHARLS). Participants were divided into four groups: those with both depressive symptoms and CVD (DS+/CVD+), those with only depressive symptoms (DS+/CVD−), those with only CVD (DS−/CVD+), and those with neither depressive symptoms nor CVD (DS−/CVD−). Additionally, stratified analyses were conducted to differentiate participants based on sleep statuses and gender.

**Results:**

In the absence of potential confounding variables, the phenotypes DS+/CVD+, DS+/CVD−, and DS−/CVD+ were each independently linked to a higher fall risk relative to the reference category DS−/CVD− (RR = 1.96, 95% CI: 1.25–3.07; RR = 1.92, 95% CI: 1.29–2.87; RR = 1.58, 95% CI: 1.03–2.42, respectively). Specifically, within the sleep sufficiency group, the DS+/CVD+, DS+/CVD−, and DS−/CVD+ phenotypes exhibited a significantly elevated risk of falls relative to the DS−/CVD− phenotype (RR = 2.23, 95% CI: 1.22–4.05; RR = 2.02, 95% CI: 1.19–3.43; RR = 1.73, 95% CI: 1.02–2.93, respectively). After adjusting for confounding variables, Males with DS−/CVD+ phenotypes are significantly more likely to fall (RR = 2.04, 95% CI: 1.04–3.98). In contrast, the DS+/CVD+ and DS+/CVD− phenotypes are linked to a heightened risk of falls in females, with relative risk of 1.79 (95% CI: 1.04–3.09) and 1.82 (95% CI: 1.11–2.98), respectively. Furthermore, there was no evidence of an additive interaction between depression and CVD in relation to fall risk among patients with T2DM (*p* > 0.05).

**Conclusion:**

The co-occurrence of depression and CVD significantly elevates the risk of falls in diabetic patients. It is recommended that female patients prioritize the prevention and management of depression, whereas male patients should focus on the prevention and management of cardiovascular diseases. Additionally, ensuring adequate sleep is essential for all patients.

## 1 Introduction

Diabetes encompasses a heterogeneous group of disorders marked by abnormalities in glucose regulation (1). T2DM is most prevalent among diabetics. The prevalence of diabetes in China is approximately 12.8%, with T2DM comprising 95% of the diabetic population (2). T2DM can result in numerous severe complications, including diabetic foot, cardiovascular disease, cerebrovascular disease, and blindness, all of which impose significant social and economic burdens. T2DM patients are more likely to fall compared to healthy individuals, according to an increasing body of research (3–5). Consequently, an effective intervention needs to be developed based on empirical evidence to address the risk factors associated with falls in diabetics.

Depression, a prevalent mental disorder globally, has a reported prevalence of 16.8% in China (6). The relationship between depression and the onset of T2DM has been studied in several studies (7–9). It is unequivocally established that depression increases the risk of T2DM (10). According to research, depression is twice as common among women as it is among men (11). Depression is linked to circadian rhythms and sleep disturbances, with women exhibiting more severe depressive symptoms also demonstrating poorer sleep continuity (12). Globally, CVD—a chronic and multifaceted condition resulting from heart and vascular disorders—remains the foremost cause of premature mortality and chronic disability (13). Atherosclerosis-related vascular disorders are also associated with diabetes, according to studies (14). Numerous studies have demonstrated an association between both the quantity and quality of sleep and the risk of CVD (15, 16). The incidence of falls escalates with advancing age, particularly among middle-aged and older adults individuals with T2DM.

The China Health and Retirement Longitudinal Study (CHARLS) is a comprehensive national panel survey targeting China’s middle-aged and older adults population, encompassing over 337 million individuals (17). While some researchers have leveraged the CHARLS datasets to explore the effects of body mass index (BMI) and grip strength on fall risk among older adults, there is a notable scarcity of research focusing specifically on the susceptibility of individuals with diabetes to falls. A cross-sectional study utilizing the CHARLS dataset examined the association between frailty and fall risk in diabetic patients but did not establish a conclusive link between frailty and falls. Although depression and cardiovascular disease are acknowledged as independent risk factors for the onset of T2DM, physiological and behavioral variations, such as sleep patterns and physical activity levels, among middle-aged and older adults may contribute to differing fall rates and outcomes.

Consequently, this study aims to investigate the combined effects of depression and CVD on fall risk in patients with T2DM, utilizing data from the CHARLS. Additionally, the study seeks to evaluate the potential moderating effects of different sleep statuses and gender on this association.

## 2 Methods

### 2.1 Participants

We utilized data from two waves of CHARLS database. The CHARLS study is a nationally representative, population-based longitudinal health survey conducted by the National School of Development at Peking University. It targets individuals aged 45 and older in China. In 2011, a baseline survey was conducted, followed by follow-up surveys in 2013, 2015, 2018, 2020 (18). Respondents were selected by means of a multistage probability sampling strategy. Data collection for the CHARLS database was ethically approved by the Biomedical Ethics Review Committee of Peking University (approval no. IRB00001052-11015). All participants in the CHARLS database provided informed consent.

A longitudinal cohort study was undertaken utilizing baseline data from the 2015 CHARLS and follow-up data from 2018. Initially, a preliminary screening of the general demographics of 21,038 participants was conducted, resulting in 1,670 individuals meeting the inclusion criteria. Participants lacking either a BMI measurement or a depression scale score (*n* = 497) were subsequently excluded. Consequently, 1,173 participants were monitored over a three-year period, with further exclusions applied to those who were lost to follow-up (*n* = 232). Ultimately, the final sample for the longitudinal cohort study comprised 941 participants.

### 2.2 Data sources

Baseline data encompassing individual characteristics, sociodemographic information, physical measurements, and both physical and mental health status were collected through a structured questionnaire. The outcome measures for falls were derived from summary data collected during the follow-up period in 2018. The exposure measures, including depressive symptoms and CVD, along with covariates such as age, BMI, sex, sleep duration, marital status, residence, education, smoking status, alcohol consumption, hypertension, dyslipidemia, and disability, were obtained from baseline data collected in 2015. In order to perform subsequent bioassays, trained nurses collected venous blood samples from fasting participants.

A self-reported questionnaire item asked: “Have you been diagnosed by your physician with dyslipidemia, hypertension, or T2DM?” evaluated hypertension, dyslipidemia, and T2DM. Participants who responded affirmatively to this question were classified as having dyslipidemia, hypertension, or T2DM (19).

CVD was assessed through self-reported diagnoses provided by participants. Specifically, participants were queried with the following questions: “Have you been diagnosed with coronary heart disease, heart attack, congestive heart failure, angina, or other heart problems by a physician?” and “Have you been diagnosed with a stroke?” Individuals who answered affirmatively to either question were classified as having CVD (20).

Sleep duration was evaluated through a self-reported questionnaire in which respondents were asked, “What is the average number of hours of sleep you got during the past month? This may be less than the number of hours you spend in bed.” Sleep insufficiency and sufficiency were operationally defined as less than 6 h and 6 h or more, respectively.

The Center for Epidemiologic Studies Depression Scale (CES-D) was employed to assess the severity of depressive symptoms in CHARLS (21). This instrument has been validated for evaluating depression within the Chinese population. There are 10 items on the CES-D, each rated on a 4-point Likert scale, asking respondents to indicate how frequently they have experienced depressive symptoms during the past week. Depression is indicated by a score of 10 or higher on the total score, which ranges from 0 to 30 (22).

### 2.3 Outcome

Using the question “Have you fallen down in the last two years?” as the primary outcome, this study assessed whether diabetic patients fall. Participants who responded affirmatively to the question regarding falls were categorized as having experienced falls.

### 2.4 Statistical analysis

Categorical variables were expressed as percentages, and the Chi-square test was employed for group comparisons of categorical variables. Continuous variables were presented as mean ± standard deviation, and comparisons were conducted using either the ANOVA or the Mann–Whitney U test.

Multivariate logistic regression analysis was employed to examine the impact of depressive symptoms and CVD on the risk of falls among diabetic patients, with the calculation of relative risk (RR) both unadjusted and adjusted. Drawing from existing literature (2), variables that demonstrated statistical significance in the univariate analysis were included as covariates in the logistic regression model. The study constructed three distinct models: Model 1 was unadjusted; Model 2 was adjusted for age, BMI, sex, sleep duration, marital status, residence, education, smoking status, and alcohol consumption; and Model 3 was further adjusted for age, BMI, sex, sleep duration, marital status, residence, education, smoking status, alcohol consumption, hypertension, dyslipidemia, and disability.

To enhance the precision of our evaluation regarding the combined association of depressive symptoms and CVD with the incidence of falls among diabetic patients, an analysis of additive interactions was conducted using the relative excess risk due to interaction (RERI), attributable proportion due to interaction (AP), and synergy index (SI) (23). In the absence of biological interaction, both RERI and AP would equal 0, while SI would equal 1.

In this study, SPSS (version 26) software was used for the statistical analysis, and R software was used for the statistical analysis (version 3.5.1 from the Vienna, Austria-based R Foundation for Statistical Computing), and GraphPad Prism software (version 8.0.2), all *p*-values are two-sided and *p* = 0.05 is considered statistically significant.

## 3 Results

### 3.1 Characteristics of baseline population

**Figure 1:**
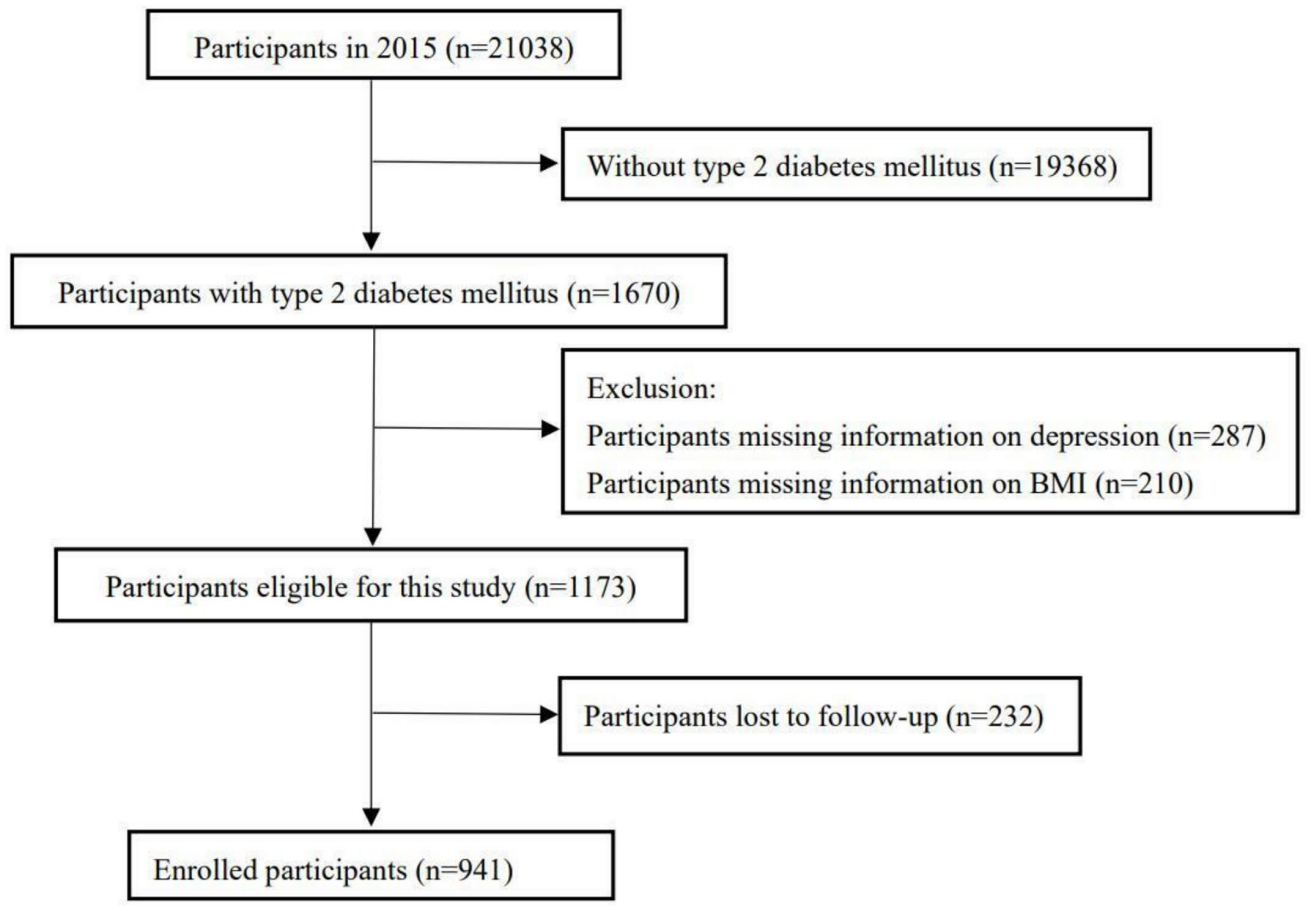
Flow chart for participants selection and follow-up.

The sample selection process is illustrated in Figure 1. A total of 941 eligible participants took part in this cohort study. Follow-up took place over a period of time, 254 new cases of falls were documented, accounting for 26.99% of the study population. Table 1 presents the baseline characteristics of the participants. The mean age of the participants was 62.27 ± 8.56 years. Additionally, 375 participants (39.85%) were identified as having depression, while 357 participants (37.94%) were diagnosed with CVD. Depressive symptoms were observed among participants in the same CVD categories (DS+/CVD+ vs. DS−/CVD+ and DS+/CVD− vs. DS−/CVD−) were significantly more likely to experience sleep insufficiency (50.00% vs. 31.47%, *p* < 0.001, and 44.65% vs. 22.22%, *p* < 0.001, respectively), be female (71.87% vs. 52.79%, *p* < 0.001, and 66.51% vs. 54.47%, *p* < 0.01), reside in rural areas (61.25% vs. 39.59%, *p* < 0.001, and 61.86% vs. 46.61%, *p* < 0.001), have a history of hypertension (53.13% vs. 42.64%, *p* < 0.05, and 39.07% vs. 34.96%, *p* < 0.05), and report disability (63.12% vs. 43.65%, *p* < 0.001, and 50.70% vs. 29.27%, *p* < 0.001) compared to those without depressive symptoms. Within the same categories of depressive symptoms, participants with CVD (DS+/CVD+ vs. DS+/CVD−, DS−/CVD+ vs. DS−/CVD−) were more likely to exhibit a higher body mass index (BMI) (25.98 ± 3.96 vs. 24.87 ± 3.68, *p* < 0.01; 26.15 ± 3.79 vs. 25.31 ± 3.40, *p* < 0.01), a history of dyslipidemia (60.63% vs. 45.12%, *p* < 0.01; 43.65% vs. 29.27%, *p* < 0.01), and disability (63.12% vs. 50.70%, *p* < 0.05; 50.70% vs. 29.27%, *p* < 0.001) compared to those without CVD. Based on the detailed baseline information provided in Table 1 and Supplementary Tables 1, 2, no significant differences were observed between these groups when it comes to marital status or smoking status.

**Table 1:** Characteristics of baseline population.

Variables	Total (*n* = 941)	DS+/CVD+ (*n* = 160)	DS+/CVD− (*n* = 215)	DS−/CVD+ (*n* = 197)	DS−/CVD− (*n* = 369)	*P*
Age, years, mean ± SD	62.27 ± 8.56	63.19 ± 7.99	61.50 ± 8.35	64.36 ± 9.15	61.21 ± 8.38	<0.001
BMI group, Kg/m2, Mean ± SD	25.50 ± 3.68	25.98 ± 3.96	24.87 ± 3.68	26.15 ± 3.79	25.31 ± 3.40	0.001
Sleep duration						<0.001
Insufficiency	320 (34.01)	80 (50.00)	96 (44.65)	62 (31.47)	82 (22.22)	
Sufficiency	621 (65.99)	80 (50.00)	119 (55.35)	135 (68.53)	287 (77.78)	
Sex, *n* (%)						<0.001
Male	378 (40.17)	45 (28.13)	72 (33.49)	93 (47.21)	168 (45.53)	
Female	563 (59.83)	115 (71.87)	143 (66.51)	104 (52.79)	201 (54.47)	
Marital status, *n* (%)						0.137
Married	829 (88.10)	136 (85.00)	185 (86.05)	172 (87.31)	336 (91.06)	
Others	112 (11.90)	24 (15.00)	30 (13.95)	25 (12.69)	33 (8.94)	
Residence, *n* (%)						<0.001
Rural	481 (51.12)	98 (61.25)	133 (61.86)	78 (39.59)	172 (46.61)	
Urban	460 (48.88)	62 (38.75)	82 (38.14)	119 (60.41)	197 (53.39)	
Education, *n* (%)						<0.001
Illiterate	399 (42.40)	92 (57.50)	112 (52.09)	62 (31.47)	133 (36.05)	
Primary school	205 (21.79)	30 (18.75)	50 (23.26)	43 (21.83)	82 (22.22)	
Middle school	199 (21.15)	26 (16.25)	36 (16.74)	44 (22.34)	93 (25.20)	
High school or above	138 (14.66)	12 (7.50)	17 (7.91)	48 (24.36)	61 (16.53)	
Smoking status, *n* (%)						0.550
No	756 (80.34)	132 (82.50)	176 (81.86)	160 (81.22)	288 (78.05)	
Yes	185 (19.66)	28 (17.50)	39 (18.14)	37 (18.78)	81 (21.95)	
Alcohol consumption, *n* (%)						0.018
No	682 (72.48)	132 (82.50)	151 (70.23)	142 (72.08)	257 (69.65)	
Yes	259 (27.52)	28 (17.50)	64 (29.77)	55 (27.92)	112 (30.35)	
Hypertension, *n* (%)						<0.001
No	559 (59.40)	75 (46.87)	131 (60.93)	113 (57.36)	240 (65.04)	
Yes	382 (40.60)	85 (53.13)	84 (39.07)	84 (42.64)	129 (34.96)	
Dyslipidemia, *n* (%)						<0.001
No	472 (50.16)	63 (39.37)	118 (54.88)	78 (39.59)	213 (57.72)	
Yes	469 (49.84)	97 (60.63)	97 (45.12)	119 (60.41)	156 (42.28)	
Disability, *n* (%)						<0.001
No	537 (57.07)	59 (36.88)	106 (49.30)	111 (56.35)	261 (70.73)	
Yes	404 (42.93)	101 (63.12)	109 (50.70)	86 (43.65)	108 (29.27)	
Falls (Yes), *n* (%)	254 (26.99)	57 (35.63)	73 (33.95)	55 (27.92)	69 (18.70)	<0.001

### 3.2 The impact of various depressive symptoms and CVD phenotypes on the incidence of falls in diabetic patients

**Figure 2:**
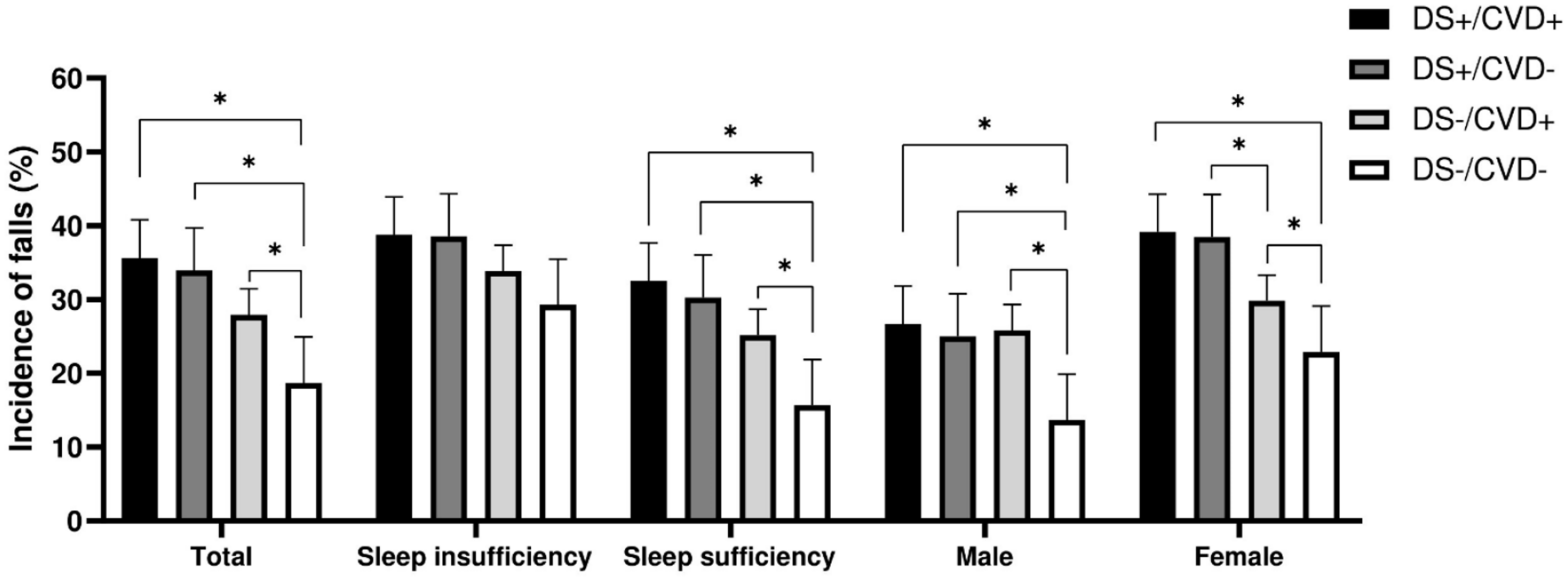
Incidence of falls across different depressive symptoms and CVD phenotypes within the two groups. *: *p* < 0.05, DS, depressive symptoms; CVD, cardiovascular disease.

During the follow-up period, a total of 254 individuals with T2DM reported a history of falls, yielding a cumulative incidence of 26.99%. We employed the Chi-square test to compare the incidence of falls across different depressive symptomatology and CVD phenotypes within the two groups (Figure 2). A significant increase in falls was observed among participants with depressive symptoms (DS+/CVD+ and DS+/CVD−) compared to participants without depressive symptoms (DS−/CVD+ and DS−/CVD−). Additionally, among individuals with depression, those with concurrent CVD demonstrated a significantly higher incidence of falls than those without CVD.

**Figure 3:**
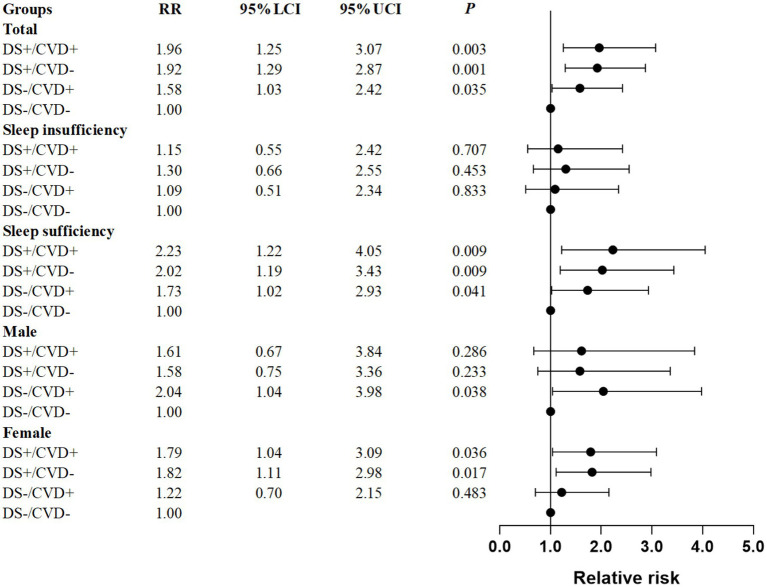
Forest plot for the relative risk of the combined association of depressive symptoms and CVD on fall risk in patients with T2DM. Relative risk are after adjustment for age, BMI, sex, sleep duration, marital status, residence, education, smoking status, alcohol consumption, hypertension, dyslipidemia, and disability. DS, depressive symptoms; CVD, cardiovascular disease.

Logistic regression was used to investigate the independent effects of various phenotypes on falls. Taking into account potential confounding factors, the phenotypes DS+/CVD+, DS+/CVD−, and DS−/CVD+ were found to be independently associated with an increased risk of falls compared to the reference category DS−/CVD− (RR = 1.96, 95% CI: 1.25–3.07; RR = 1.92, 95% CI: 1.29–2.87; RR = 1.58, 95% CI: 1.03–2.42, respectively) (Figure 3; Table 2).

**Table 2:** Results of logistic regression analysis for combined association of depressive symptoms and CVD on fall risk in patients with T2DM.

Groups	N	Falls (%)	Model 1		Model 2		Model 3	
			RR (95%CI)	*P*	RR (95%CI)	*P*	RR (95%CI)	*P*
Total	941	26.99						
DS+/CVD+	160	35.63	2.41 (1.59–3.65)	<0.001	2.19 (1.42–3.37)	<0.001	1.96 (1.25–3.07)	0.003
DS+/CVD−	215	33.95	2.24 (1.52–3.29)	<0.001	2.06 (1.39–3.06)	<0.001	1.92 (1.29–2.87)	0.001
DS−/CVD+	197	27.92	1.68 (1.12–2.53)	0.012	1.64 (1.08–2.50)	0.021	1.58 (1.03–2.42)	0.035
DS−/CVD−	369	18.7	Reference		Reference		Reference	
Sleep insufficiency	320	35.31						
DS+/CVD+	80	38.75	1.53 (0.79–2.94)	0.204	1.26 (0.63–2.51)	0.512	1.15 (0.55–2.42)	0.707
DS+/CVD−	96	38.54	1.52 (0.81–2.84)	0.195	1.37 (0.71–2.65)	0.351	1.30 (0.66–2.55)	0.453
DS−/CVD+	62	33.87	1.24 (0.61–2.52)	0.555	1.01 (0.48–2.14)	0.981	1.09 (0.51–2.34)	0.833
DS−/CVD−	82	29.27	Reference		Reference		Reference	
Sleep sufficiency	621	22.71						
DS+/CVD+	80	32.5	2.41 (1.47–4.56)	0.001	2.51 (1.40–4.50)	0.002	2.23 (1.22–4.05)	0.009
DS+/CVD−	119	30.25	2.33 (1.41–3.86)	0.001	2.20 (1.31–3.70)	0.003	2.02 (1.19–3.43)	0.009
DS−/CVD+	135	25.19	1.81 (1.10–2.99)	0.021	1.88 (1.12–3.15)	0.017	1.73 (1.02–2.93)	0.041
DS−/CVD−	287	15.68	Reference		Reference		Reference	

Model 1: was unadjusted; Model 2: was adjusted for age, BMI, sex, sleep duration, marital status, residence, education, smoking status, and alcohol consumption; Model 3: was further adjusted for age, BMI, sex, sleep duration, marital status, residence, education, smoking status, alcohol consumption, hypertension, dyslipidemia, and disability.

### 3.3 Sleep duration and sex contribute to the risk of falls in diabetic patients across different phenotypes

For patients with sufficient sleep, the DS+/CVD+, DS+/CVD−, and DS−/CVD+ phenotypes demonstrated a higher incidence of falls compared to the DS−/CVD− phenotype (32.50, 30.25, 25.19% vs. 15.68%, *p* < 0.05). Contrary to what was observed in patients with inadequate sleep, there was no significant difference between these phenotypes (Table 2; Figure 2). Furthermore, logistic regression analysis was conducted to adjust for potential confounders. Within the sleep sufficiency group, the DS+/CVD+, DS+/CVD−, and DS−/CVD+ phenotypes were associated with a significantly increased risk of falls compared to the DS−/CVD− phenotype (RR = 2.23, 95% CI: 1.22–4.05; RR = 2.02, 95% CI: 1.19–3.43; RR = 1.73, 95% CI: 1.02–2.93, respectively) (Table 2; Figure 3).

Given the sex-specific differences in both CVD and depressive symptoms, we performed a sex-stratified analysis. Among males, the incidence of falls was significantly higher in the DS+/CVD+, DS+/CVD−, and DS−/CVD+ phenotypes compared to the DS−/CVD− phenotype (26.67, 25.00, 25.81% vs. 13.69%, *p* < 0.05). Similarly, among females, those with depressive symptoms (DS+/CVD+ and DS+/CVD−) exhibited a higher incidence of falls compared to the DS−/CVD− phenotype (39.13, 38.46% vs. 22.89%, *p* < 0.05) (refer to Table 3 and Figure 2). Notably, after adjusting for confounding variables, it was found that the DS−/CVD+ phenotype is associated with an increased risk of falls exclusively in males (RR = 2.04, 95% CI: 1.04–3.98). Conversely, the DS+/CVD+ and DS+/CVD− phenotypes are associated with a heightened risk of falls exclusively in females (RR = 1.79, 95% CI: 1.04–3.09 and RR = 1.82, 95% CI: 1.11–2.98, respectively) (refer to Table 3 and Figure 2).

**Table 3:** Results of logistic regression analysis for combined association of depressive symptoms and CVD on fall risk in patients with T2DM stratified by sex.

Groups	*N*	Falls (%)	Model 1		Model 2		Model 3	
			RR (95%CI)	*P*	RR (95%CI)	*P*	RR (95%CI)	*P*
Male	378	20.37						
DS+/CVD+	45	26.67	2.29 (1.04–5.07)	0.041	1.96 (0.85–4.52)	0.113	1.61 (0.67–3.84)	0.286
DS+/CVD−	72	25.00	2.10 (1.05–4.20)	0.035	1.82 (0.87–3.82)	0.111	1.58 (0.75–3.36)	0.233
DS−/CVD+	93	25.81	2.19 (1.16–4.16)	0.016	2.17 (1.12–4.19)	0.022	2.04 (1.04–3.98)	0.038
DS−/CVD−	168	13.69	Reference		Reference		Reference	
Female	563	31.44						
DS+/CVD+	115	39.13	2.17 (1.32–3.57)	0.002	1.99 (1.18–3.36)	0.010	1.79 (1.04–3.09)	0.036
DS+/CVD−	143	38.46	2.11 (1.32–3.37)	0.002	1.95 (1.20–3.17)	0.007	1.82 (1.11–2.98)	0.017
DS−/CVD+	104	29.81	1.43 (0.84–2.44)	0.188	1.28 (0.73–2.24)	0.385	1.22 (0.70–2.15)	0.483
DS−/CVD−	201	22.89	Reference		Reference		Reference	

Model 1: was unadjusted; Model 2: was adjusted for age, BMI, sex, sleep duration, marital status, residence, education, smoking status, and alcohol consumption; Model 3: was further adjusted for age, BMI, sex, sleep duration, marital status, residence, education, smoking status, alcohol consumption, hypertension, dyslipidemia, and disability.

### 3.4 Depressive symptoms and CVD are associated with a greater risk of falls in diabetics

To evaluate the influence of depressive symptoms and CVD on the risk of falling, we calculated the Relative Excess Risk due to Interaction (RERI), Attributable Proportion (AP), and Synergy Index (SI) (Table 4). The results indicated no evidence of additive interaction (RERI = −0.534, 95% CI: −1.740 to 0.672; AP = −0.221, 95% CI: −0.755 to 0.313; SI = 0.726, 95% CI: 0.360 to 1.464). Additionally, no multiplicative interaction was observed (*p* > 0.05, Supplementary Table 3). Stratified analyses by different sleep durations and sex yielded consistent results.

**Table 4:** Quantitative analysis of additive interaction between depressive symptoms and CVD on fall risk in patients with T2DM.

Groups	SI	95%CI	AP	95%CI	RERI	95%CI
Total	0.726	0.360–1.464	−0.221	−0.755-0.313	−0.534	−1.740–0.672
Sleep insufficiency	0.671	0.121–3.711	−0.175	−1.027-0.677	−0.272	−1.580–1.036
Sleep sufficiency	0.742	0.290–1.894	−0.214	−0.946-0.518	−0.554	−2.297–1.190
Male	0.563	0.147–2.164	−0.437	−1.609-0.735	−1.002	−3.318–1.314
Female	0.745	0.296–1.875	−0.185	−0.823-0.453	−0.404	−1.734-0.926

## 4 Discussion

Globally, 463 million people live with the disease in 2019 (24). Projections indicate that this figure will escalate to 700 million by 2045 (25). T2DM represents a substantial global health burden, significantly impacting individuals’ quality of life. Our study revealed that 26.99% of diabetic patients experienced falls during the follow-up period, with 51.15% of these patients also suffering from depression and 44.09% from CVD. In middle-aged and older adults Chinese patients with T2DM with depression and CVD, this study represents the first attempt to analyze their combined effect on fall risk, with analyses stratified by sleep status and gender. It appears that both depression and CVD increase the risk of falls in diabetics, which is consistent with previous studies (26). Notably, the concomitance of depression and CVD may exacerbate fall risk in diabetic patients more than the presence of either condition alone, particularly among women. Further analysis of the individual and combined effects of depression and CVD on fall risk revealed no evidence of additive or multiplicative interactions between these conditions. Compared to the DS−/CVD− phenotype, the DS+/CVD+, DS+/CVD−, and DS−/CVD+ phenotypes were generally identified as risk factors for T2DM. Notably, the risk of falls in the DS+/CVD+ group was approximately 1.96 times higher than that in the DS−/CVD− group. There was no significant association between depression and falls in the meta-analysis (27). In order to better understand depression’s impact on fall risk among diabetics, further research is needed.

Our findings indicate that CVD can elevate the risk of diabetes, corroborating previous studies (28). CVD complications, such as sarcopenia, are significant risk factors for falls (29). Additionally, Aged individuals with elevated levels of cardiac troponin T and N-terminal pro-B-type natriuretic peptide (NT-pro BNP) are more likely to fall (30). Our findings indicate that CVD can elevate the risk of diabetes, corroborating previous research (30). Additionally, Depression and falls seem to be bidirectionally correlated, according to studies. Depressive disorders are frequently associated with excessive fear of falling, which further increases the likelihood of falling. Cognitive, sensory, and motor pathways all play a role in the association between depression and fear of falling and disturbances in gait and balance (31). Furthermore, the use of antidepressants has been shown to elevate the risk of falls (32). Depressive symptoms can hinder patients’ adherence to medical recommendations, such as timely medication intake, weight management, and increased physical activity, potentially leading to falls in individuals with diabetes (33). However, Depression and falls continue to be a controversial topic.

The concomitance of depressive symptoms and CVD may elevate the incidence of falls among patients with T2DM. Previous studies have demonstrated that both depressive symptoms and CVD can serve as independent risk factors for falls (34–37). Furthermore, the coexistence of depression and CVD can directly or indirectly influence the onset and progression of diabetes, thereby augmenting the risk of falls. However, our study revealed that depressive symptoms did not exhibit any additive interaction with CVD in relation to falls in patients with T2DM. Limited research has explored this interaction among middle-aged and older adults Chinese populations, highlighting the need for further investigation. It is noteworthy that individuals with diabetes are particularly susceptible to other chronic systemic diseases. In this study, we observed that the coexistence of one or more chronic diseases with depression or cardiovascular disease heightened the risk of falls in diabetic patients. This suggests that these factors play a significant role, which is not only associated with the clinical manifestations of the diseases but may also be linked to the chronic stress resulting from the prolonged duration of depression and cardiovascular disease, as well as the potential exacerbation of these conditions. Such chronic stress may be intricately connected to the combined effects of economic pressure, mental strain, physical health, psychological state, and the disease burden experienced by diabetic patients. Therefore, the management of chronic disease comorbidities in diabetic patients should be prioritized in future research and healthcare strategies.

Given that depressive symptoms and CVD typically exhibit gender-specific variations, Analyses were conducted based on gender. Among men, the DS−/CVD+ phenotype emerged as a significant risk factor for falls in diabetic patients when compared to the DS−/CVD− phenotype, whereas neither the DS+/CVD+ nor the DS+/CVD− phenotypes demonstrated a similar association. In female patients, a comparison of the DS+/CVD+ and DS+/CVD− phenotypes and DS−/CVD− phenotype revealed that both were significantly associated with falls, whereas the DS−/CVD+ phenotype was not. Patients with female diagnoses had a higher rate of falls than patients with male diagnoses. Women with diabetes are at greater risk of falling than men with diabetes due to depression, while men with diabetes are at greater risk of falling due to CVD. This study primarily included women who were either perimenopausal or postmenopausal. During this stage, women experience substantial physical and psychological changes due to the decline in ovarian function (38). These women are more likely to suffer from depressive symptoms than their older counterparts, potentially attributable to an elevated risk of metabolic disorders stemming from age-related alterations in steroidogenesis, which may contribute to the incidence of falls (39). In addition to influencing glucose and lipid metabolism, sex hormones and sex-specific molecular mechanisms have also been found to affect cardiac energy metabolism, the prevalence of CVD in men is higher than that in women (40). This disparity may account for the higher incidence of falls in male patients.

The analysis based on sleep duration indicated that the incidence of falls in the sleep insufficiency group was 35.31%, suggesting that sleep insufficiency constitutes a risk factor for falls among diabetic patients. In the sleep insufficiency cohort, the DS+/CVD+, DS+/CVD−, and DS−/CVD+ phenotypes did not emerge as significant risk factors for falls among diabetic individuals when compared to the DS−/CVD− phenotype. Conversely, within the cohort receiving adequate sleep, these three phenotypes were significantly associated with an increased risk of falls relative to the DS−/CVD− phenotype. Consequently, it can be inferred that sleep deprivation markedly elevates the incidence of falls in diabetic patients, surpassing the impact of DS and CVD factors. Previous research has established that poor sleep quality heightens fall risk (41, 42), and insufficient sleep has been linked to an increased incidence of sarcopenia, thereby further elevating fall risk (43).

This study constitutes the inaugural examination of the synergistic impact of depression and CVD on fall risk in patients with T2DM within this specific demographic in China. Additionally, it evaluates the interplay between various sleep statuses and gender on this combined effect. The strengths of our investigation include a substantial sample size sourced from a national cohort study, which significantly bolsters the robustness and generalizability of our findings. The findings of this study may serve as a valuable reference for the prevention of falls among diabetic patients in China. Implementing targeted interventions to enhance the quality of life and alleviate physical symptoms in middle-aged and older diabetic individuals of varying genders could potentially mitigate the risk of falls. Despite existing research, substantial gaps remain, requiring further studies to clarify the mechanisms behind falls in diabetic patients.

Diabetes patients with falls are at high risk for falls, and this study holds significant potential as a reference for preventing and treating falls. It is crucial for patients with T2DM to prioritize the management of depression and CVD in order to mitigate the associated risk of falls. However, our study is subject to several limitations. Firstly, the data on T2DM, depression, and other medical conditions were obtained through self-reported questionnaires, which may introduce biases in the assessment of these conditions. Secondly, we did not consider the potential effects of medication and the length of illness in our analysis. Lastly, the study was unable to incorporate critical factors contributing to falls in diabetic patients, such as peripheral neuropathy. Consequently, future research should aim to undertake more extensive and comprehensive prospective cohort studies with extended follow-up durations to substantiate our findings.

## 5 Conclusion

In summary, our study determined that the concurrent presence of depression and CVD substantially elevates the risk of falls among diabetic patients, a finding that is particularly relevant to middle-aged and older adults populations. However, no significant interaction between depression and CVD on fall risk was observed in this cohort. The prevention and management of depression should be emphasized for female patients, whereas for male patients, priority should be given to the prevention and management of CVD. Patients should ensure adequate sleep and maintain a healthy lifestyle.

## Data Availability

The datasets presented in this study can be found in online repositories. The names of the repository/repositories and accession number(s) can be found below: https://charls.charlsdata.com/.
