# Supplementary material for: The impact of depression and cardiovascular disease on fall risk in type 2 diabetes mellitus: a gender and sleep status analysis
Source: Front Public Health. 2025 Mar 19;13:1488923. doi: 10.3389/fpubh.2025.1488923 (PMC11963775; doi:10.3389/fpubh.2025.1488923)
Supplement: Supplementary file 1 [file Table_1.DOCX]

Supplemental Table 1 Characteristics of baseline population (DS+/CVD+ vs. DS-/CVD+; DS+/CVD- vs. DS-/CVD-)

| **Variables** | **Total**  **(n = 941)** | **DS+/CVD+**  **(n = 160)** | **DS-/CVD+**  **(n = 197)** | ***P*** | **DS+/CVD-**  **(n = 215)** | **DS-/CVD-**  **(n = 369)** | ***P*** |
| --- | --- | --- | --- | --- | --- | --- | --- |
| Age, years, Mean ±SD | 62.27±8.56 | 63.19±7.99 | 64.36±9.15 | 0.203 | 61.50±8.35 | 61.21±8.38 | 0.688 |
| BMI group, Kg/m2, Mean ±SD | 25.50±3.68 | 25.98±3.96 | 26.15±3.79 | 0.607 | 24.87±3.68 | 25.31±3.40 | 0.143 |
| Sleep duration  Insufficiency  sufficiency | 320(34.01)  621(65.99) | 80(50.00)  80(50.00) | 62(31.47)  135(68.53) | <0.001 | 96(44.65)  119(55.35) | 82(22.22)  287(77.78) | <0.001 |
| Sex, n (%)  Male  Female | 378(40.17)  563(59.83) | 45(28.13)  115(71.87) | 93(47.21)  104(52.79) | <0.001 | 72(33.49)  143(66.51) | 168(45.53)  201(54.47) | 0.004 |
| Marital status, n (%)  Married  Others | 829(88.10)  112(11.90) | 136(85.00)  24(15.00) | 172(87.31)  25(12.69) | 0.528 | 185(86.05)  30(13.95) | 336(91.06)  33(8.94) | 0.060 |
| Residence, n (%)  Rural  Urban | 481(51.12)  460(48.88) | 98(61.25)  62(38.75) | 78(39.59)  119(60.41) | <0.001 | 133(61.86)  82(38.14) | 172(46.61)  197(53.39) | <0.001 |
| Education, n (%)  Illiterate  Primary school  Middle school  High school or above | 399(42.40)  205(21.79)  199(21.15)  138(14.66) | 92(57.50)  30(18.75)  26(16.25)  12(7.50) | 62(31.47)  43(21.83)  44(22.34)  48(24.36) | <0.001 | 112(52.09)  50(23.26)  36(16.74)  17(7.91) | 133(36.05)  82(22.22)  93(25.20)  61(16.53) | <0.001 |
| Smoking status, n (%)  No  Yes | 756(80.34)  185(19.66) | 132(82.50)  28(17.50) | 160(81.22)  37(18.78) | 0.755 | 176(81.86)  39(18.14) | 288(78.05)  81(21.95) | 0.272 |
| Alcohol consumption, n (%)  No  Yes | 682(72.48)  259(27.52) | 132(82.50)  28(17.50) | 142(72.08)  55(27.92) | 0.020 | 151(70.23)  64(29.77) | 257(69.65)  112(30.35) | 0.882 |
| Hypertension, n (%)  No  Yes | 559(59.40)  382(40.60) | 75(46.87)  85(53.13) | 113(57.36)  84(42.64) | 0.048 | 131(60.93)  84(39.07) | 240(65.04)  129(34.96) | 0.320 |
| Dyslipidemia, n (%)  No  Yes | 472(50.16)  469(49.84) | 63(39.37)  97(60.63) | 78(39.59)  119(60.41) | 0.966 | 118(54.88)  97(45.12) | 213(57.72)  156(42.28) | 0.504 |
| Disability, n (%)  No  Yes | 537(57.07)  404(42.93) | 59(36.88)  101(63.12) | 111(56.35)  86(43.65) | <0.001 | 106(49.30)  109(50.70) | 261(70.73)  108(29.27) | < 0.001 |
| Falls, (Yes), n (%) | 254(26.99) | 57(35.63) | 55(27.92) | 0.119 | 73(33.95) | 69(18.70) | <0.001 |

Supplemental Table 2 Characteristics of baseline population (DS+/CVD+ vs. DS+/CVD-; DS-/CVD+ vs. DS-/CVD-)

| **Variables** | **Total**  **(n = 941)** | **DS+/CVD+**  **(n = 160)** | **DS+/CVD-**  **(n = 215)** | ***P*** | **DS-/CVD+**  **(n = 197)** | **DS-/CVD-**  **(n = 369)** | ***P*** |
| --- | --- | --- | --- | --- | --- | --- | --- |
| Age, years, Mean ±SD | 62.27±8.56 | 63.19±7.99 | 61.50±8.35 | 0.050 | 64.36±9.15 | 61.21±8.38 | <0.001 |
| BMI group, Kg/m2, Mean ±SD | 25.50±3.68 | 25.98±3.96 | 24.87±3.68 | 0.006 | 26.15±3.79 | 25.31±3.40 | 0.008 |
| Sleep duration  Insufficiency  sufficiency | 320(34.01)  621(65.99) | 80(50.00)  80(50.00) | 96(44.65)  119(55.35) | 0.305 | 62(31.47)  135(68.53) | 82(22.22)  287(77.78) | 0.016 |
| Sex, n (%)  Male  Female | 378(40.17)  563(59.83) | 45(28.13)  115(71.87) | 72(33.49)  143(66.51) | 0.268 | 93(47.21)  104(52.79) | 168(45.53)  201(54.47) | 0.703 |
| Marital status, n (%)  Married  Others | 829(88.10)  112(11.90) | 136(85.00)  24(15.00) | 185(86.05)  30(13.95) | 0.775 | 172(87.31)  25(12.69) | 336(91.06)  33(8.94) | 0.161 |
| Residence, n (%)  Rural  Urban | 481(51.12)  460(48.88) | 98(61.25)  62(38.75) | 133(61.86)  82(38.14) | 0.904 | 78(39.59)  119(60.41) | 172(46.61)  197(53.39) | 0.109 |
| Education, n (%)  Illiterate  Primary school  Middle school  High school or above | 399(42.40)  205(21.79)  199(21.15)  138(14.66) | 92(57.50)  30(18.75)  26(16.25)  12(7.50) | 112(52.09)  50(23.26)  36(16.74)  17(7.91) | 0.706 | 62(31.47)  43(21.83)  44(22.34)  48(24.36) | 133(36.05)  82(22.22)  93(25.20)  61(16.53) | 0.150 |
| Smoking status, n (%)  No  Yes | 756(80.34)  185(19.66) | 132(82.50)  28(17.50) | 176(81.86)  39(18.14) | 0.873 | 160(81.22)  37(18.78) | 288(78.05)  81(21.95) | 0.377 |
| Alcohol consumption, n (%)  No  Yes | 682(72.48)  259(27.52) | 132(82.50)  28(17.50) | 151(70.23)  64(29.77) | 0.006 | 142(72.08)  55(27.92) | 257(69.65)  112(30.35) | 0.545 |
| Hypertension, n (%)  No  Yes | 559(59.40)  382(40.60) | 75(46.87)  85(53.13) | 131(60.93)  84(39.07) | 0.007 | 113(57.36)  84(42.64) | 240(65.04)  129(34.96) | 0.072 |
| Dyslipidemia, n (%)  No  Yes | 472(50.16)  469(49.84) | 63(39.37)  97(60.63) | 118(54.88)  97(45.12) | 0.003 | 78(39.59)  119(60.41) | 213(57.72)  156(42.28) | <0.001 |
| Disability, n (%)  No  Yes | 537(57.07)  404(42.93) | 59(36.88)  101(63.12) | 106(49.30)  109(50.70) | 0.016 | 111(56.35)  86(43.65) | 261(70.73)  108(29.27) | 0.001 |
| Falls, (Yes), n (%) | 254(26.99) | 57(35.63) | 73(33.95) | 0.737 | 55(27.92) | 69(18.70) | 0.012 |

Supplemental Table 3 Quantitative analysis of multiplicative interaction between depressive symptoms and CVD on fall risk in patients with T2DM

| **Variables** | **OR** | **95%LCI** | **95%LCI** | **P** |
| --- | --- | --- | --- | --- |
| Depressive symptoms | 2.26 | 1.53 | 3.32 | <0.001 |
| CVD | 1.69 | 1.13 | 2.54 | 0.011 |
| Depressive symptoms & CVD | 0.63 | 0.35 | 1.14 | 0.129 |
